# Protective Efficiency Comparison of Direct and Remote Ischemic Preconditioning on Ischemia Reperfusion Injury of the Liver in Patients Undergoing Partial Hepatectomy

**DOI:** 10.1155/2023/2763320

**Published:** 2023-01-07

**Authors:** Erliang Kong, Chang Yuan, Yongchang Li, Tian Tian, Yan He, Xudong Feng

**Affiliations:** ^1^Department of Anesthesiology, The 988th Hospital of Joint Logistic Support Force of Chinese People's Liberation Army, Zhengzhou, Henan 450042, China; ^2^Department of Anesthesiology, The First Affiliated Hospital of Zhengzhou University, Zhengzhou, Henan 450052, China; ^3^Department of Anesthesiology, Changzheng Hospital, Second Affiliated Hospital of Naval Medical University, Shanghai 200003, China; ^4^Department of Anesthesiology, Fuzhou Maternity and Child Health Care Hospital, Fuzhou, 350000 Fujian, China

## Abstract

**Objective:**

Ischemia reperfusion injury greatly damages liver function and deteriorates the prognosis of patients undergoing partial hepatectomy. This study is to compare the protective efficiency of direct and remote ischemic preconditioning (DIPC and RIPC) on ischemia reperfusion injury of the liver in patients undergoing partial hepatectomy.

**Methods:**

90 patients scheduled for partial hepatectomy were enrolled and randomly divided into control (*n* = 30), DIPC (*n* = 30), and RIPC (*n* = 30) groups. Baseline and surgery characteristics were collected, and ischemic preconditioning methods were carried out. Intraoperative hemodynamics, liver function and liver reserve capacity, oxidative stress, and inflammatory responses were measured, and the incidence of postoperative adverse reactions was calculated finally.

**Results:**

10 patients were excluded from the study, and finally, the eligible patients in three groups were 27, 28, and 25, separately. No significant differences were observed in baseline and surgery characteristics among the three groups. SBP and DBP were significantly higher after hepatic portal vein occlusion while they were significantly lower after surgery in the DIPC and RIPC groups compared with that in the control group, SBP and DBP were of great fluctuation at different time points in the control group while they showed much more stabilization in the DIPC and RIPC groups. ALT, AST, and TBIL were significantly decreased on days 1, 3, and 5 after surgery, and ICG R15 was significantly decreased while ICG K value and EHBF were significantly increased on day 1 after surgery in the DIPC and RIPC groups compared with that in the control group. Moreover, antioxidant enzyme SOD was increased, and inflammatory factors TNF-*α* and IL-1*β* were decreased 24 hours after surgery in the DIPC and RIPC groups compared with that in the control group. DIPC and RIPC also decreased hospital stays and the incidence of nausea, vomiting, and hypertension.

**Conclusion:**

DIPC and RIPC both alleviated ischemia reperfusion injury of the liver and reduced perioperative complications with similar protective efficiency in patients undergoing partial hepatectomy.

## 1. Introduction

Hepatic carcinoma is one of the most common malignant tumors worldwide. Clinically, partial hepatectomy is still the most effective surgical treatment for hepatic carcinoma, liver abscess, liver cyst, or other diseases [1]. As the hepatic portal vein provides nearly 70% blood flow for the liver, surgeons usually provisionally block the hepatic portal vein during hepatectomy to reduce bleeding, resulting in hepatic ischemia reperfusion injury simultaneously [2]. Ischemia reperfusion injury refers to the sudden restoration of blood supply to organs or tissues after a period of severe ischemia, resulting in the substantial damage and dysfunction [3]. Ischemia reperfusion injury of the liver causes great damages to perioperative liver function, which seriously affect the outcomes and prognosis in patients underwent liver transplantation or hepatectomy. After the regaining of blood supply, the ischemia liver shows aggravated dysfunction and structural damages, even facilitates liver failure [4]. With the deepening understanding of this phenomenon, the pathophysiological process of hepatic ischemia reperfusion injury has been paid more attention. Effective prevention of ischemia reperfusion injury caused by hepatic portal vein blocking has become a vital factor to improve the success rate of hepatectomy.

Recently, various studies focus on the protective effects of ischemic preconditioning on organs. The concept of direct ischemic preconditioning (DIPC) was first proposed by Murry et al. in 1986; it was confirmed to inhibit the secretion of inflammatory factors and reactive oxygen species (ROS) through stimulating the release of adenosine, nitric oxide, or other substances directly, which could strengthen the capacity in withstanding injury [5]. A prospective controlled clinical trial conducted by Clavien et al. found that DIPC alleviated ischemia reperfusion injury of the liver, accompanied by the increased risk of prolonging operation and ischemia time [6]. Subsequently, Przyklenk et al. found that remote ischemic preconditioning (RIPC) could also affect the conditions of distant organs through neural or humoral mechanisms [7]. The remote tissues or organs pretreated with RIPC may produce endogenous opioids, adenosine, catecholamines, or other neurotransmitters and release into the blood to improve the tolerance of remote organs to injury. Some studies found that RIPC reduced the adhesion of neutrophils and improved the oxygen incorporation of mitochondria in hepatocytes and reduce the production of acid substances, thereby alleviating the inflammatory reaction and oxidative damages [8]. RIPC also increased red blood cell velocity, reduced cell swelling, alleviated accumulation of harmful products, and improved cellular energy metabolism to improve stress tolerance of organ [9]. Therefore, RIPC provides a new idea for the protection of organ ischemia reperfusion injury.

This study focused on comparing the protective efficiency of DIPC and RIPC on ischemia reperfusion injury of the liver in patients undergoing partial hepatectomy. We evaluated the changes of intraoperative hemodynamics, liver function, liver reserve capacity, oxidative stress, and inflammatory responses 24 hours after surgery and postoperative adverse reactions. These results may help to explore new strategies in alleviating hepatic ischemia reperfusion injury with fewer perioperative complications.

## 2. Materials and Methods

### 2.1. Subjects

This prospective, double-blinded, randomized controlled clinical trial was conducted to evaluate the protective efficiency of DIPC and RIPC on ischemia reperfusion injury of the liver in patients undergoing partial hepatectomy. We used the CONSORT 2010 checklist when writing our report [10]. This study was approved by the Committee on Ethics of Biomedicine of the 988th Hospital of Joint Logistic Support Force of Chinese People's Liberation Army (20210015) and registered in the Chinese Clinical Trial Center (ChiCTR2200064608). Enrolled patients were well evaluated by the study group and acquired detailed information about the study. Patients meeting the inclusion criteria (30-70 years old, primary liver cancer with size less than 10 cm, American Society of Anesthesiologists (ASA) classification I-III, hepatic Child-Pugh classification A-B, scheduled for elective hepatic surgery under general anesthesia for the first time, and standard hepatectomy with Pringle's method to block the hepatic portal vein during surgery) were enrolled in this study with written permission. Patients with peripheral vascular disease affecting the upper limb function, severe liver cirrhosis, total hepatic portal vein blocking duration more than 60 minutes, bleeding volume more than 1000 mL, history of heart infarction, cerebral infarction, diabetes, endocrine diseases, opioid addiction or chronic obstructive pulmonary disease, and using of corticosteroids and other anti-inflammatory drugs were excluded from the study.

### 2.2. Grouping

**Figure 1:**
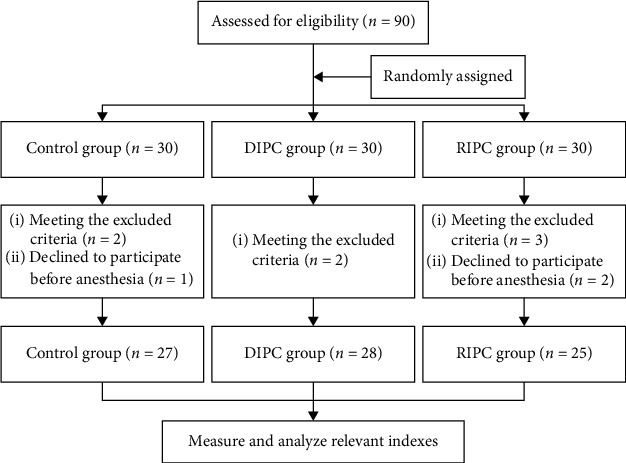
Flow diagram of grouping.

As the flow diagram shown in Figure 1, 90 patients were enrolled and randomly divided into control (*n* = 30), DIPC (*n* = 30), and RIPC (*n* = 30) groups. Two met the excluded criteria, and 1 declined to participate before anesthesia in the control group; 2 met the excluded criteria in the DIPC group, 3 met the excluded criteria, and 2 declined to participate before anesthesia in the RIPC group, and finally, the eligible patients in three groups were 27, 28, and 25, separately. Baseline and surgery characteristics in perioperative period of patients were collected. The double-blinded measures were conducted by sealing the number of patients in opaque envelopes that were opened by a well-trained nurse before induction of general anesthesia. Each envelope contained the group allocation with instructions for the ischemic preconditioning method. Operators were blinded to the patients' group allocation. Patients and data analyzers were also blinded to the group assignment.

### 2.3. Procedural Protocol

Before anesthesia, patients in three groups received invasive arterial blood pressure, oxygen saturation, and electrocardiogram monitoring routinely. Anesthesia induction was performed by midazolam 0.04 mg/kg, sufentanil 0.5 *μ*g/kg, propofol 4 mg/kg, and cisatracurium 0.3 mg/kg intravenously; then, the endotracheal tube was placed correctly. Anesthesia maintenance was performed by propofol 40 *μ*g/(kg·min), cisatracurium 0.1 mg/(kg·h) continuously, and sufentanil 0.25 *μ*g/kg at 30-minute intervals intravenously. Patients in the control group received no measurement; patients in the DIPC group received DIPC by blocking the hepatic portal vein for 5-minute ischemia and 5-minute reperfusion for three cycles before partial hepatectomy; patients in the RIPC group received RIPC by blocking the blood flow of the right upper limb with an automatic pressure tourniquet (ATS-I, Germany) inflated to 26 kPa for 5-minute ischemia and 5-minute reperfusion for three cycles before surgery. The vital signs of the patients were maintained stably during the surgery, and the endotracheal tube was removed after the patient was fully awake.

### 2.4. Liver Reserve Capacity Measurements

Indocyanine green (ICG) clearance test was carried out to quantify the capacity of remaining functional hepatocytes by detecting the clearance ability of the liver to ICG metabolism, biotransformation, and excretion, which can be used to assess the liver reserve capacity dynamically [11]. ICG retention ratio after 15 minutes (ICG R15), ICG plasma clearance rate (ICG K value), and effective hepatic blood flow (EHBF) were calculated to reflect the liver reserve capacity [12]. ICG clearance tests were carried out by DDG-3300K analyzer (Optoelectronic Industry, Japan). Patients were asked to fast for 6 hours before test, and the nasal photosensitive probe of the analyzer was placed in the nasal alar; then, the prepared ICG solution was injected uniformly through the median cubital vein within 10 seconds. DDG data analysis software was used to automatically analyzed ICG R15, ICG K value, and EHBF.

### 2.5. Outcomes

The primary outcomes were liver function indexes (alanine transaminase (ALT), aspartate aminotransferase (AST), and total bilirubin (TBIL)), liver reserve capacity (ICG R15, ICG K value, and EHBF), and oxidative stress and inflammatory responses 24 hours after surgery (superoxide dismutase (SOD), tumor necrosis factor *α* (TNF-*α*), and interleukin 1*β* (IL-1*β*)) to evaluate the liver injury and inflammatory condition. The second outcomes were baseline (age, sex, body mass index (BMI), ASA, hemoglobin, Child-Pugh grading, and mean arterial pressure (MAP)) and surgery characteristics (surgery duration, hepatic portal vein occlusion duration, bleeding volume, urine volume, infusion volume, propofol consumption, and sufentanil consumption) in perioperative period and intraoperative hemodynamics (systolic blood pressure (SBP), diastolic blood pressure (DBP), and heart rate) before anesthesia (T0), 5 minutes after induction (T1), 5 minutes after laparotomy (T2), 5 minutes after hepatic portal vein occlusion (T3), and after surgery (T4). Incidence of postoperative adverse reactions (respiratory depression, nausea, vomiting, hypotension, and hypertension) and hospital stays were also calculated after surgery. A more than 20% increase or decrease in MAP was regarded as hypertension or hypotension. Hitachi 7180 automatic biochemical analyzer (HITACHI, Japan) was used to measure blood biochemical indexes.

### 2.6. Statistical Analysis

To compare the protective efficiency of DIPC and RIPC on ischemia reperfusion injury of the liver with a statistical significance, we calculated the sample size with a 50% reduction in the ALT level in the DIPC and RIPC groups compared with the control group according to previous studies [13]. By using *α* = 0.05 and 1 − *β* = 0.8, we calculated that 78 patients were required, and finally, we enrolled 90 patients. All data was analyzed using GraphPad Prism 9 Software (San Diego, CA, USA) and received the normality test. Quantitative data were expressed as mean ± standard deviation. One-way ANOVA was performed to compare data among all groups followed by Bonferroni's test. Categorical data was expressed by percentage followed by the chi-square test. *P* < 0.05 was considered statistically significant.

## 3. Results

### 3.1. Baseline Characteristics of Patients Undergoing Partial Hepatectomy

As shown in Table 1, patients in three groups showed no significant differences in age, sex, BMI, and ASA classification (all *P* > 0.05). Also, there were no significant differences in hemoglobin, Child-Pugh grading, and MAP among the three groups (all *P* > 0.05).

### 3.2. Surgery Characteristics in Perioperative Period

Additionally, no significant differences were displayed in surgery duration, hepatic portal vein occlusion duration, bleeding volume, urine volume, infusion volume, propofol consumption, and sufentanil consumption among the three groups in perioperative period (all *P* > 0.05) (Table 2).

### 3.3. Comparison of Intraoperative Hemodynamics

Further, we explored the effects of DICP and RICP on the intraoperative hemodynamics at different time points in perioperative period. As shown in Table 3, the SBP, DBP, and heart rate in three groups showed no statistic differences at T0, T1, and T2 (all *P* > 0.05). However, SBP and DBP were significantly higher at T3 while they were significantly lower at T4 in the DIPC and RIPC groups compared with that in the control group (all *P* < 0.05). The heart rate was significantly lower at T3 and T4 in the DIPC and RIPC groups compared with that in the control group (all *P* < 0.05). Moreover, SBP and DBP were of great fluctuation at different time points in the control group while they showed much more stabilization in the DIPC and RIPC groups. No statistic differences in SBP, DBP, and heart rate at different time points were observed between the DIPC and RIPC groups.

### 3.4. Changes of Liver Function after Surgery

Next, we compared the protective efficiency of DIPC and RIPC on ischemia reperfusion injury of the liver in patients undergoing partial hepatectomy by evaluating the liver function and liver reserve capacity. As shown in Table 4, ALT, AST, TBIL, ICG R15, ICG K value, and EHBF in three groups all showed no statistic differences before surgery (all *P* > 0.05). However, ALT, AST, and TBIL were significantly decreased on days 1, 3, and 5 after surgery in the DIPC and RIPC groups compared with that in the control group (all *P* < 0.05), suggesting that both DIPC and RIPC could alleviate hepatocyte injury in ischemia reperfusion injury of the liver after surgery. Also, ICG R15 was significantly decreased while ICG K value and EHBF were significantly increased on day 1 after surgery in the DIPC and RIPC groups compared with that in the control group (all *P* < 0.05), suggesting that both DIPC and RIPC could improve liver reserve capacity in ischemia reperfusion injury of the liver. But no statistic differences in ALT, AST, TBIL, ICG R15, ICG K value, and EHBF were observed between the DIPC and RIPC groups.

### 3.5. Changes of Oxidative Stress and Inflammatory Responses 24 Hours after Surgery

We further explored whether ischemic preconditioning alleviated oxidative stress and inflammatory responses in ischemia reperfusion injury of the liver. As shown in Table 5, DIPC and RIPC significantly increased the antioxidant enzyme SOD and decreased the inflammatory factors TNF-*α* and IL-1*β* 24 hours after surgery compared that in the control group (all *P* < 0.05), suggesting that both DIPC and RIPC could alleviate oxidative stress and inflammatory responses in ischemia reperfusion injury of the liver. No statistic differences in SOD, TNF-*α*, and IL-1*β* were observed between the DIPC and RIPC groups.

### 3.6. Incidence of Postoperative Adverse Reactions

Finally, we observed the incidence of postoperative adverse reactions to evaluate the disadvantages of ischemic preconditioning. As shown in Table 6, the incidence of nausea, vomiting, and hypertension was significantly lower in the DIPC and RIPC groups compared with that in the control group (all *P* < 0.05). DIPC and RIPC also decreased hospital stays of patients (*P* < 0.05).

## 4. Discussion

Clinically, one of the major challenges to successful partial hepatectomy is ischemia reperfusion injury of the liver, and it is urgent to seek for new noninvasive methods to alleviate hepatic ischemia reperfusion injury [14]. The present study found that both DIPC and RIPC improved liver function and liver reserve capacity with similar protective efficiency through weakening oxidative stress and inflammatory responses in patients undergoing partial hepatectomy. Moreover, DIPC and RIPC acquired much more stable intraoperative hemodynamics and postoperative adverse reactions, which was promising in improving the outcomes and prognosis of patients undergoing partial hepatectomy.

The mechanism of hepatic ischemia reperfusion injury caused by hepatic portal vein occlusion during partial hepatectomy is relatively complex and mainly involves ROS accumulation, cell apoptosis, inflammatory reactions, and other pathological processes, which seriously damages the normal physiological function of hepatocytes [15]. During ischemia, the activities of ROS scavenger SOD weaken and the production of ROS gradually increases, resulting in the imbalance of redox reaction. Moreover, the acid metabolite accumulation further damages the antioxidant system and induces DNA damage and lipid peroxidation [16]. Excessive consumption of ATP in hepatocytes in ischemic environment results in the inhibition of Na^+^/K^+^ ion channels in mitochondria and the increase of mitochondrial membrane permeability for uncontrollable calcium influx [17]. Meanwhile, ischemia and hypoxia also damages the function of electron transport chain and thereby induces ROS accumulation, which further damages the mitochondrial membrane and activates caspase-3 mediating apoptosis pathway [18]. After reperfusion of blood flow, excessive inflammatory factors and ROS are released into the blood flow, resulting in a cascade of inflammatory reactions and the dysfunction of hepatocytes or adjacent tissues [19]. Therefore, it is of great significance to explore effective and noninvasive strategies to alleviate hepatic ischemia reperfusion injury after hepatic portal vein occlusion by inhibiting hepatic peroxidation, apoptosis, inflammation, or other pathological processes.

Ischemic preconditioning has been confirmed to be a safe and efficient method with potential protective effect on organs recently. Studies have confirmed that ischemic preconditioning can protect liver function in hepatic ischemia reperfusion injury by inhibiting hepatocyte apoptosis, inducing autophagy, or activating related signaling pathways [20, 21]. RIPC is characterized by handling remote limbs or tissues instead of directly handling the target organs, which is easy to operate clinically. Some studies implied that three cycles of 5-minute ischemia and 5-minute reperfusion significantly decreased troponin I level in cardiac surgery and protected myocardial cells [22, 23]. RIPC can affect the stress response of distant organs through neural and humoral mechanisms. The distal tissues or organs pretreated with RIPC produce endogenous opioids, adenosine, catecholamines, or other neurotransmitters to interact with the intracellular PKC pathway in hepatocytes, thereby reducing calcium overload and improving the tolerance of hepatocytes to injury [24]. Meanwhile, tissues or organs can also release humoral regulatory factors and act on hepatocytes to regulate liver stress responses through intercellular junctions [25]. Sheng et al. found that RIPC reduced the adhesion of liver neutrophils and the production of acid substances in rats, which was beneficial to alleviating inflammatory responses and oxidative damage [26]. Tomschi et al. found that RIPC reduced hepatocyte edema, inflammation, and microcirculation damage by increasing red blood cell velocity, reducing neutrophil adhesion and cell death in rats [27]. Here, we also confirmed that both DIPC and RIPC decreased the ALT, AST, and TBIL levels on days 1, 3, and 5 after surgery. Also, DIPC and RIPC decreased ICG R15 and increased ICG K value and EHBF on day 1 after surgery. DIPC and RIPC even decreased the incidence of postoperative adverse reactions such as nausea, vomiting, and hypertension. These all supported the conclusion that ischemic preconditioning could alleviate liver injury and improve liver reserve capacity in ischemia reperfusion injury of the liver with much more stable intraoperative hemodynamics. However, no significant differences were observed between DIPC and RIPC pretreatment, but RIPC showed some superiority than DIPC in conveniences and efficiency in alleviating liver injury and improving liver reserve capacity.

Oxidation reaction and inflammatory responses are surely activated in hepatic ischemia reperfusion injury [28, 29]. Massive proinflammatory factors such as TNF-*α*, IL-6, and IL-1*β* are released in the microenvironment of the liver. The damaged antioxidant system also contributes to hepatocyte injury and inflammatory responses [30]. Thus, the antioxidant enzymes and proinflammatory factors may be the indicators of liver injury. Alleviating the progression of oxidation reaction and inflammatory responses can effectively protect the normal function of hepatocytes [31]. This study also tested the oxidative stress and inflammatory responses and found that DIPC and RIPC both significantly increased the antioxidant enzyme SOD and decreased inflammatory factors TNF-*α* and IL-1*β* 24 hours after surgery, further confirming that ischemic preconditioning could protect liver function through preventing the progression of oxidative stress and inflammatory responses in ischemia reperfusion injury.

## 5. Conclusions

In summary, both DIPC and RIPC alleviated ischemia reperfusion injury of the liver and reduced perioperative complications with similar protective efficiency in patients undergoing partial hepatectomy, and RIPC acquired some superiority than DIPC in conveniences and efficiency. These results may help to provide new strategies in alleviating hepatic ischemia reperfusion injury.

## Figures and Tables

**Table 1:** Baseline characteristics of patients undergoing partial hepatectomy.

Characteristics	Control group (*n* = 27)	DIPC group (*n* = 28)	RIPC group (*n* = 25)	*P* value
Age (years)	53.81 ± 12.13	54.32 ± 10.68	54.48 ± 9.77	0.974
Sex (male/female)	17/10	15/13	16/9	0.688
BMI (kg/m^2^)	24.37 ± 2.64	23.57 ± 2.68	22.98 ± 3.26	0.215
ASA (%)				0.908
I	10 (37.04)	8 (28.57)	9 (36.00)	
II	12 (44.44)	15 (53.57)	13 (52.00)	
III	5 (18.52)	5 (17.86)	3 (12.00)	
Hemoglobin (g/dL)	11.45 ± 1.80	11.56 ± 1.28	11.35 ± 1.48	0.876
Child-Pugh grading (%)				0.475
A	16 (59.26)	12 (42.86)	13 (52.00)	
B	11 (40.74)	16 (57.14)	12 (48.00)	
MAP (mmHg)	83.63 ± 12.45	84.11 ± 11.87	86.60 ± 12.05	0.642

**Table 2:** Surgery characteristics in perioperative period.

Characteristics	Control group (*n* = 27)	DIPC group (*n* = 28)	RIPC group (*n* = 25)	*P* value
Surgery duration (min)	146.93 ± 25.56	143.89 ± 31.57	146.16 ± 26.25	0.917
Hepatic portal vein occlusion duration (min)	40.96 ± 7.93	39.82 ± 8.84	42.24 ± 6.79	0.544
Bleeding volume (mL)	546.67 ± 89.05	525.36 ± 95.94	541.20 ± 80.74	0.656
Urine volume (mL)	494.44 ± 130.07	498.21 ± 100.30	460.80 ± 120.52	0.455
Infusion volume (mL)	1992.59 ± 268.59	1932.14 ± 290.66	2032.00 ± 306.49	0.449
Propofol consumption (mg)	892.96 ± 124.52	906.07 ± 123.45	877.20 ± 114.04	0.688
Sufentanil consumption (*μ*g)	56.44 ± 7.83	57.57 ± 10.22	60.20 ± 8.79	0.314

**Table 3:** Comparison of intraoperative hemodynamics.

Groups	Hemodynamics	Time points				
		T0	T1	T2	T3	T4
Control group (*n* = 27)	SBP (mmHg)	139.30 ± 10.33	98.26 ± 10.01	120.93 ± 20.22	103.30 ± 19.17	131.22 ± 18.14
	DBP (mmHg)	90.22 ± 9.23	63.70 ± 6.73	81.04 ± 13.48	72.41 ± 14.84	79.37 ± 8.12
	Heart rate	84.63 ± 9.72	65.22 ± 10.16	74.85 ± 10.20	76.81 ± 10.86	81.70 ± 12.33

DIPC group (*n* = 28)	SBP (mmHg)	134.04 ± 11.72	102.14 ± 10.79	117.25 ± 11.95	112.86 ± 10.64^∗^	114.36 ± 11.49^∗^
	DBP (mmHg)	85.32 ± 8.45	64.29 ± 9.43	77.07 ± 8.87	69.96 ± 6.98^∗^	67.64 ± 10.45^∗^
	Heart rate	83.57 ± 10.86	66.54 ± 12.07	72.32 ± 10.65	65.21 ± 8.18^∗^	70.18 ± 13.77^∗^

RIPC group (*n* = 25)	SBP (mmHg)	136.72 ± 13.30	104.72 ± 8.04	112.56 ± 12.00	109.92 ± 8.87^∗^	111.12 ± 7.97^∗^
	DBP (mmHg)	83.36 ± 12.04	65.96 ± 7.55	69.32 ± 12.77	64.44 ± 8.08^∗^	66.52 ± 8.37^∗^
	Heart rate	81.16 ± 16.08	68.40 ± 13.54	69.4 ± 14.83	66.40 ± 9.36^∗^	72.52 ± 15.67^∗^

^∗^ *P* < 0.05 compared with the control group.

**Table 4:** Changes of liver function after surgery.

Groups	Liver function	Days after surgery			
		-1	1	3	5
Control group (*n* = 27)	ALT (U/L)	78.61 ± 9.23	459.38 ± 67.01	368.48 ± 87.30	212.15 ± 56.35
	AST (U/L)	99.48 ± 16.74	525.46 ± 71.64	435.26 ± 72.59	262.92 ± 68.92
	TBIL (*μ*mol/L)	34.01 ± 10.89	71.17 ± 11.85	49.46 ± 9.62	33.31 ± 7.47
	ICG R15 (%)	12.31 ± 3.84	19.29 ± 3.91	15.15 ± 4.03	13.85 ± 4.15
	ICG K value (/min)	0.24 ± 0.04	0.17 ± 0.05	0.21 ± 0.05	0.22 ± 0.05
	EHBF (L/min)	1.07 ± 0.27	0.50 ± 0.13	0.74 ± 0.24	0.99 ± 0.21

DIPC group (*n* = 28)	ALT (U/L)	82.29 ± 13.59	359.35 ± 48.86^∗^	305.24 ± 62.96^∗^	138.05 ± 60.61^∗^
	AST (U/L)	90.50 ± 18.27	455.95 ± 61.28^∗^	380.86 ± 61.17^∗^	197.18 ± 51.34^∗^
	TBIL (*μ*mol/L)	37.88 ± 9.37	53.81 ± 12.89^∗^	40.39 ± 9.56^∗^	22.96 ± 3.26^∗^
	ICG R15 (%)	12.63 ± 4.05	16.63 ± 3.37^∗^	13.60 ± 3.65	13.31 ± 2.98
	ICG K value (/min)	0.26 ± 0.05	0.19 ± 0.05^∗^	0.23 ± 0.06	0.24 ± 0.06
	EHBF (L/min)	1.15 ± 0.37	0.64 ± 0.24^∗^	0.80 ± 0.22	1.26 ± 0.38

RIPC group (*n* = 25)	ALT (U/L)	89.19 ± 17.54	322.18 ± 52.72^∗^	289.64 ± 68.39^∗^	111.64 ± 40.15^∗^
	AST (U/L)	95.71 ± 19.34	442.67 ± 78.36^∗^	358.07 ± 72.60^∗^	173.06 ± 57.13^∗^
	TBIL (*μ*mol/L)	35.67 ± 14.11	50.38 ± 10.69^∗^	37.78 ± 7.95^∗^	21.65 ± 7.11^∗^
	ICG R15 (%)	11.42 ± 3.77	15.90 ± 3.24^∗^	14.65 ± 2.74	13.03 ± 4.53
	ICG K value (/min)	0.26 ± 0.07	0.21 ± 0.06^∗^	0.23 ± 0.07	0.25 ± 0.07
	EHBF (L/min)	1.06 ± 0.34	0.77 ± 0.27^∗^	0.82 ± 0.27	1.31 ± 0.40

^∗^ *P* < 0.05 compared with the control group.

**Table 5:** Changes of oxidative stress and inflammatory responses 24 hours after surgery.

Characteristics	Control group (*n* = 27)	DIPC group (*n* = 28)	RIPC group (*n* = 25)	*P* value
SOD (U/mL)	109.00 ± 19.43	156.44 ± 38.51	179.05 ± 34.09	0.001
TNF-*α* (ng/L)	166.73 ± 44.33	141.44 ± 37.29	135.20 ± 35.77	0.011
IL-1*β* (ng/L)	270.51 ± 55.60	235.43 ± 52.65	232.78 ± 60.76	0.028

**Table 6:** Incidence of postoperative adverse reactions.

Adverse reactions	Control group (*n* = 27)	DIPC group (*n* = 28)	RIPC group (*n* = 25)	*P* value
Respiratory depression (%)	3 (11.11)	3 (10.71)	2 (8.00)	0.921
Nausea (%)	10 (37.04)	4 (14.29)	3 (12.00)	0.047
Vomiting (%)	8 (29.63)	3 (10.71)	1 (4.00)	0.026
Hypotension (%)	2 (7.41)	2 (7.14)	1 (4.00)	0.854
Hypertension (%)	12 (44.44)	6 (21.43)	3 (12.00)	0.032
Hospital stays (days)	12.41 ± 3.15	10.50 ± 2.96	10.40 ± 2.24	0.017

## Data Availability

The datasets used and analyzed during the current study are available from the corresponding authors on reasonable request.
